# A case report involving suppressed nuclear receptor transcription factors 4a1 and Stevens-Johnson syndrome induced by a single dose of pembrolizumab and successfully treated with early steroid administration, resulting in complete remission of stage III lung cancer

**DOI:** 10.1186/s40780-022-00261-y

**Published:** 2022-12-05

**Authors:** Maiko Machida, Chika Yamazaki, Nao Kouda, Yousei Hanai, Hideki Sato, Ainari Konda, Yuka Yamagata, Tatsuya Itho, Haruhiko Aisaka

**Affiliations:** 1 Department of Pharmacotherapy, Faculty of Pharmaceutical Sciences, Hokkaido University of Science, 7-15-4-1 Maeda Teine, Sapporo, Hokkaido 006-8585 Japan; 2 Department of Pharmacy and Respiratory Medicine, Japan Community Health Care Organization Sapporo Hokushin Hospital, 2-6-2-1 Atsubetsu Chou Atsubetsu, Sapporo, Hokkaido 004-8618 Japan

**Keywords:** Pembrolizumab, Stevens-Johnson syndrome, Nuclear receptor transcription factor 4a

## Abstract

**Background:**

Immunotherapy with immune checkpoint inhibitors is associated with immune-related adverse events (irAEs). A positive correlation between treatment efficacy and irAEs has been reported. Clinical indicators are required for appropriate interventions, such as steroid administration, to prevent fatal outcomes. Nuclear receptor transcription factor 4a (Nr4a), which is involved in T-cell anergy, exhaustion, and regulatory T cells, were observed not only in thymocytes but in peripheral blood mononuclear cells. We describe a case of Stevens-Johnson syndrome (SJS) that was induced by a single dose of pembrolizumab and successfully treated with steroids, leading to complete remission of lung cancer during the monitoring of immune response indices, including *Nr4a1* mRNA.

**Case presentation:**

A 68-year-old male with squamous cell lung cancer (cT2aN3M0, stage IIIb) received a single dose of pembrolizumab (200 mg). On Day 21 of treatment, SJS appeared, and the patient was treated with prednisolone 60 mg/day, which was gradually tapered off. After the disappearance of the SJS symptoms, complete remission of cancer was achieved and was maintained for more than 1 year. Acute increases in the plasma IFN-γ and IL-17 concentrations and a decrease in IL-10 concentrations were observed at the onset of SJS. Simple regression analysis showed that these changes in IL-17, IFN-γ and IL-10 were significantly influenced by the decreased expression of *Nr4a1* mRNA. The pembrolizumab levels and prednisolone doses significantly influenced the suppression of *Nr4a1* mRNA levels. Although *Nr4a1* mRNA levels in the current case fluctuated during the observation period, they were significantly lower than those in a nonresponding progressive-disease case, as well as a pembrolizumab-responding case with non-SJS but similar background. The suppression of Nr4a1 in current case, might result in upregulation of cytotoxic T cells and a reduction in functional regulatory T cells, promoting favorable antitumor immunity.

**Conclusion:**

The immune responses involving Nr4a1 suppression might relate to complete remission of lung cancer in this case, despite causing SJS, which may be attributed to synergistic effects from pembrolizumab treatment and intervention with steroids. The current case indicates the preliminarily clinical benefit of evaluating Nr4a expression-related indices as the possible clinical covariates and may serve as a milestone for appropriate future chemotherapy interventions.

**Supplementary Information:**

The online version contains supplementary material available at 10.1186/s40780-022-00261-y.

## Background

An immune checkpoint inhibitor (ICI) is an antibody against programmed cell death protein 1 that exerts an antitumor effect by reactivating antitumor immunity, and immunotherapy based on ICI has been amazingly developed [1, 2]. During ICI therapy, a systemic immune-related adverse event (irAE) due to an excessive immune response appears [3]. In addition, a modest but reproducible positive correlation between efficacy and the severity of irAEs attributed to pembrolizumab in melanoma cases has been reported [4]. Clinical indicators are required for the decision to discontinue treatment or appropriate therapeutic interventions, such as steroid administration, to prevent fatal outcomes. Recently, basal levels of the neutrophil-to-lymphocyte ratio (NLR) and platelet-to-lymphocyte ratio (PLR) have been suggested to be predictive factors for serious irAEs in patients with lung cancer [5, 6]. However, more detailed clinical indicators in terms of efficacy and side effects are needed to identify appropriate interventions for subtle conditions presented by patients during chemotherapy.

In chronic inflammatory diseases, such as cancer, T-cell exhaustion is observed, leading to the expression of inhibitory receptors and progressive loss of T-cell functions [7]. There is a need for a versatile immune responsive index in peripheral blood that can assess the effects of both inflammatory effector T cells and regulatory T-cell (Treg) development and exhaustion responses. Nuclear receptor transcription factors 4a (Nr4a) family expression is rapidly induced not only in thymocytes but also in peripheral mature T cells, following engagement of the T-cell receptor (TCR) by stimulation of a peptide fragment of antigen [8, 9], which is involved in T-cell anergy, exhaustion, and Treg maintenance [10]. Nr4a1 controls CD8^+^ T-cell development through transcriptional suppression of RUNX family transcription factor 3 (Runx3) [11]. Deletion of Nr4a increases the number of functional CD8^+^ T cells responsible for antitumor immunity, which is desirable for immunotherapy of cancer [9], while Tregs that suppress excessive antitumor immunity are reduced in irAE. Monitoring of immunoreactivity indices such as rapid reactive cytokines and Nr4a will provide clinically important insights into both efficacy and side effects.

Monitoring total ICI levels would be ideal but is technically less versatile [12]. The clinical significance of measuring plasma concentrations of unbound ICI is reported to be unclear, but since ICI disappearance varies greatly among individuals at late stages [13], we performed concomitant measurements of immune responsive indices and unbound pembrolizumab measurement using a simple method to alternatively observe its disappearance. As indices, quick reactive cytokines and target mRNAs, including Nr4a, and quantitative changes indicate involvement in the differentiation and activation of cytotoxic CD8^+^ T cells, CD4^+^ Tregs and Th17 cells [10] and would provide new findings.

We report the case of a patient with a single dose of pembrolizumab-induced Stevens-Johnson syndrome (SJS) successfully treated with steroids, resulting in complete remission of lung cancer, with monitoring immune response indices including Nr4a mRNA in peripheral blood mononuclear cells (PBMCs) and concentrations of unbound pembrolizumab in blood.

## Case presentation

The patient was a 68-year-old male with squamous cell lung cancer (cT2aN3M0, stage IIIb). Since a rapid exacerbation was observed, ICI treatment with pembrolizumab 200 mg was changed from 28 days after the one cycle of previous treatment with carboplatin AUC6 and nab-paclitaxel 100 mg/m^2^. On Day 21 of the therapy, the patient discontinued the treatment because of the onset of SJS with dermatological symptoms such as diffuse cutaneous erythema and hyperkeratotic scales, painful stomatitis and lip erythema scales, mucosal ocular symptoms and glans swelling, without any infection. The case of SJS was successfully treated with steroid therapy with prednisolone 60 mg po, dexamethasone oral ointment and fluorometholone instillation, starting from Day 21 and slowly tapering off until on Day 77. After the remission of dermatological symptoms, a clinical diagnostic CT scan revealed that the cancer in the upper right lobe and mediastinum had disappeared. Although only a single dose of pembrolizumab induced SJS (successfully treated with steroids), this therapy resulted in complete remission of stage IIIb lung cancer, which was maintained for over a year.

Laboratory tests showed negative results for hepatotoxicity, endocrine toxicity and pancreatitis during the treatment. As immune responsive indices, those involved in the differentiation and activation of cytotoxic CD8^+^ T cells, CD4^+^ Treg and Th17, were selected [10]. During hospitalization and after discharge, 9 points of blood samples were evaluated. As shown below, cytokines such as interferon-γ (IFN-γ), transforming growth factor-β (TGF-β), IL-6, IL-10, IL-17, IL-23, and IL-27 were screened for the indices, while IL-2 and IL-4 were undetected. Transcription factors such as *Nr4a1, Nr4a2,* forkhead box protein P 3 (*Foxp3*)*,* retinoic acid receptor-related orphan receptor-γt (*RORγt*)*,* lymphocyte activation gene 3 (*Lag3*)*, Runx3*, and receptor of IL-23 (*IL-23R*), which specifically expresses activated Th17 involved with Nr4a2 [14], were selected as mRNAs. Assessment of the protein levels of transcription factors involves more ethical and technical challenges and has not been performed in current cases posing difficulties. Plasma concentrations of cytokines were measured by a Legend Max human ELISA kit (BioLegend, San Diego, CA). The plasma concentration of unbound pembrolizumab was evaluated with pharmacokinetic ELISA (MBS, San Diego, CA). Total RNA was extracted from PMBCs with a Paxgene Blood RNA kit (QIAGEN, Hilden Germany). The expression of target mRNA was analyzed by quantitative real-time RT‒PCR using a 7500 Fast Real-Time PCR system (Applied Biosystems, Foster City, CA) and a One-Step TB Green PrimeScript PLUS RT‒PCR kit (Takara Bio, Shiga, Japan). Relative mRNA expression was normalized to hypoxanthine–guanine phosphoribosyl transferase (HPRT) using the 2^−△△CT^ method. The primer sequences used in this study are described in Supplementary Table 1.

**Fig. 1:**
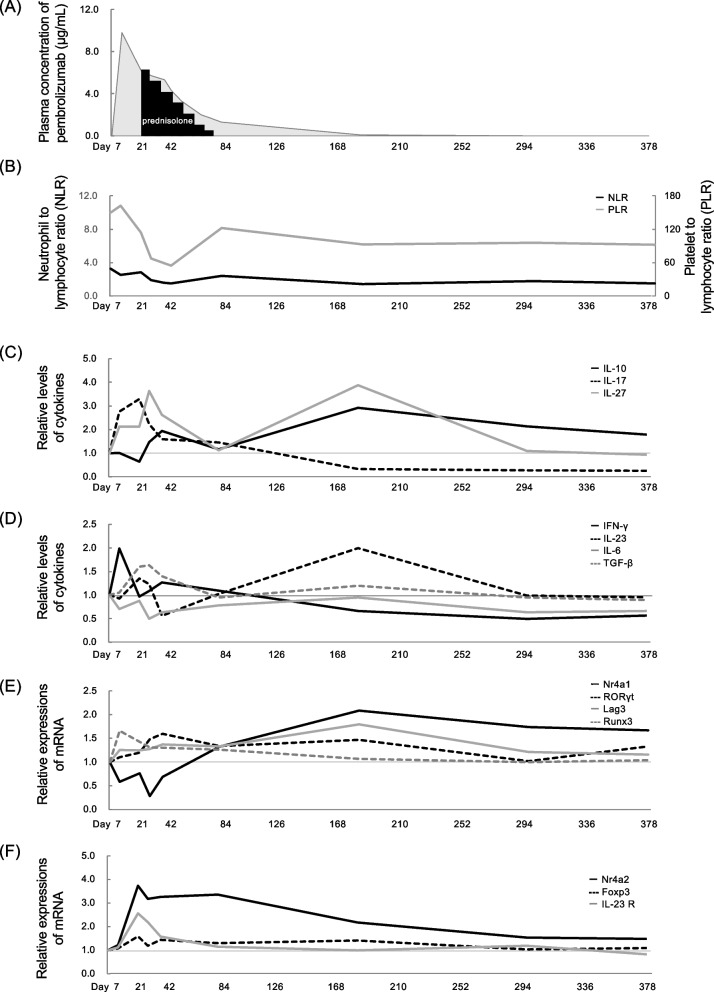
**A-F** Changes in clinical valuables during the observation periods. **A** Plasma concentration of pembrolizumab (■), administered at a dose of 200 mg by intravenous infusion on Day 0, and dose of oral prednisolone (■), started at 60 mg/day from Day 21 and slowly tapered off until Day 77. **B** Changes in the neutrophil-to-lymphocyte ratio and platelet-to-lymphocyte ratio. **C** Relative plasma cytokine concentrations. The black line indicates IL-10, the black dotted line indicates IL-17, and the gray line indicates IL-27. **D** Relative plasma cytokine concentrations. The black line indicates IFN-γ, the black dotted line indicates IL-23, the gray line indicates IL-6, and the gray dotted line indicates TGF-β. **E** Relative expression of mRNAs in peripheral blood mononuclear cells. The black line indicates *Nr4a1* mRNA, the black dotted line indicates *RORγt* mRNA*,* the gray line indicates *Lag3* mRNA, and the gray dotted line indicates *Runx3* mRNA. **F** Relative expression of mRNAs in peripheral blood mononuclear cells. The black line indicates *Nr4a2* mRNA, the black dotted line indicates *Fox3* mRNA, and the gray line indicates IL-23 receptor *(IL-23R)* mRNA

Plasma unbound pembrolizumab concentrations remained above the limit of quantification, 1.25 μg/mL, until Day 77, when prednisolone was tapered off (Fig. 1A). The pretreatment basal values for NLR, PLR, eosinophil and lymphocyte counts were 3.3, 150, 138/µL and 1.4 × 10^3^/µL, respectively. During these periods, NLR decreased gradually, and PLR increased slightly on Day 7, decreased until Day 42, and then increased (Fig. 1B).

As shown in the relative levels of cytokines (Fig. 1C, D), IL-17 increased from Day 7, peaked on Day 21, and then decreased; IL-10 decreased from Day 7, peaked on Day 21, and then increased further on Day 77; IFN-γ increased rapidly on Day 7 and then decreased; IL-23, IL-27 and TGF-β increased in a bimodal manner, peaking on Days 21 and 175; and IL-6 remained low, near the basal levels.

As shown in the relative expression of mRNAs (Fig. 1E, F), *Nr4a1* mRNAs decreased on Day 7 and remained below basal levels until Day 77. *Nr4a2* mRNAs increased from Day 7, peaked on Day 21 and remained above basal levels throughout the entire period. *Runx3* mRNAs increased on Day 7, and then were ameliorated. *Foxp3* mRNA and *IL-23R* increased on Day 21 and then were ameliorated. *RORγt* mRNA increased from Day 28. *Lag3* mRNA increased from Day 7 and peaked on Day 175, and unbound plasma pembrolizumab concentrations were detected until Day 77.

Each immune responsiveness index was statistically examined by simple regression analysis based on the individual significant analysis of variance of less than 0.05 and with a coefficient of determination greater than 0.5 (JMP Pro 16, SAS institution, Inc., Cary, NC). As shown in Table 1, IL-10 was an inhibitory independent variable, while IFN-γ and *IL-23R* mRNA were promotive independent variables, showing a significantly stronger effect on the induction of IL-17 as the dependent variable; IL-17 was an inhibitory independent variable, and *Lag3* mRNA was a promotive independent variable showing a significantly stronger effect individually on the induction of IL-10 as a dependent variable; IL-27 was a promotive independent variable showing a stronger effect on the induction of *Lag3* mRNA as a dependent variable; *Runx3* mRNA was a promotive independent variable, showing a significantly stronger effect on the induction of IFN-γ as a dependent variable. IL-10 was an inhibitory independent variable showing a significantly stronger effect on the increase in NLR; *RORγt* mRNA was an inhibitory independent variable showing a significantly stronger effect on the increase in PLR; no statistical relationship was found between IFN-γ, IL-17 and IL-10 and upstream regulators such as IL-23 and Il-27.

**Table 1:** Simple regression analysis of the pembrolizumab level and the immune responsive indices, such as blood levels of pembrolizumab, cytokines and target mRNAs for *IL-23 receptor (IL-23R), Foxp3, RORγt, Lag3* and *Runx3*

Dependent variables	Independent variables	Unstandardized coefficient	df	R^2^	F	*P* values
IL-17	IFN-γ	2.56	1, 8	0.50	6.9	0.034^*^
	IL-6	-2.27	1, 8	0.01	0.1	0.822
	IL-10	-12.20	1, 8	0.54	8.1	0.025^*^
	IL-23	-0.07	1, 8	0.01	0.1	0.785
	IL-27	0.65	1, 8	0.06	10.7	0.527
	*IL-23R* mRNA	41.57	1, 8	0.56	8.9	0.020^*^
	*RORγt* mRNA	-0.66	1, 8	0.00	0.0	0.991
	*Foxp3* mRNA	66.92	1, 8	0.17	1.5	0.264
	Pembrolizumab	8.30	1, 8	0.78	20.9	0.004^**^
IFN-γ	IL-23	-0.07	1, 8	0.12	1.0	0.355
	IL-27	0.12	1, 8	0.03	0.2	0.686
	*Runx3* mRNA	38.55	1, 8	0.60	10.3	0.015^*^
	*Lag3* mRNA	-4.75	1, 8	0.01	0.1	0.769
	Pembrolizumab	2.30	1, 8	0.77	20.6	0.004^**^
IL-10	IFN-γ	-0.11	1, 8	0.26	2.5	0.159
	IL-17	-0.04	1, 8	0.54	8.2	0.025^*^
	IL-27	0.06	1, 8	0.16	1.3	0.285
	*Foxp3* mRNA	0.06	1, 8	0.00	0.0	0.986
	*Lag3* mRNA	0.01	1, 8	0.53	7.8	0.027^*^
	Pembrolizumab	-0.35	1, 8	0.40	4.0	0.094
*Foxp3* mRNA	*Runx3* mRNA	0.51	1, 8	0.23	2.0	0.197
	Pembrolizumab	0.01	1, 8	0.01	0.1	0.807
*Lag3* mRNA	IL-27	0.03	1, 8	0.53	7.8	0.027^*^
	Pembrolizumab	-0.016	1, 8	0.08	0.5	0.496
*RORγt* mRNA	IL-6	-0.04	1, 8	0.06	0.4	0.565
	*Foxp3* mRNA	0.59	1, 8	0.32	3.3	0.112
	Pembrolizumab	0.00	1, 8	0.01	0.0	0.870
*IL-23R* mRNA	Pembrolizumab	0.08	1, 8	0.25	2.0	0.204
*Runx3* mRNA	Pembrolizumab	0.04	1, 8	0.84	30.5	0.002^**^
NLR	IL-10	-0.28	1, 8	0.71	17.1	0.004^**^
PLR	*RORγt* mRNA	-122.7	1, 8	0.58	9.8	0.017^*^

Plasma concentrations of pembrolizumab and cytokines, such as major IL-17, IFN-γ, IL-10, and those upstream of IL-6, IL-23 and IL-27, were evaluated by ELISA methods. Target mRNAs were evaluated by RT‒qPCR methods with total RNA extracted from peripheral blood mononuclear cells. The neutrophil-to-lymphocyte ratio (NLR) and platelet-to-lymphocyte ratio (PLR) were used as predictive factors for serious immune-related adverse events. Regarding these factors, only the indices extracted as a significant independent variable for these factors are presented. *p < 0.05, **P < 0.001

As shown in Table 1, the plasma concentration of pembrolizumab as an independent variable had a very strong promotive effect on the dependent variables, such as IL-17, IFN-γ and *Runx3* mRNA.

As shown in Table 2, *Nr4a1* mRNA was an inhibitory independent variable, with a very strong effect on the induction of IL-17 as a dependent variable and a strong effect on the induction of IFN-γ as a dependent variable. *Nr4a1* mRNA was a promotive independent variable, with a strong effect on the induction of IL-10 as a dependent variable; *Nr4a2* mRNA was a promotive independent variable, with individually strong effects on the induction of *Foxp3* mRNA and *IL-23R* mRNA as dependent variables. *Nr4a2* mRNA was a promotive independent variable and represented a strong effect on the induction of *Foxp3* mRNA and *IL-23R* mRNA separately as dependent variables. The plasma concentration of pembrolizumab and dose of prednisolone, as independent variables, had a very strong inhibitory effect on the induction of *Nr4a1* mRNA. For NLR and PLR, no separate statistical relationship was found between *Nr4a1* and *2* mRNA.

**Table 2:** Simple regression analysis of the pembrolizumab level, prednisolone dose, predictive factors for serious immune-related adverse events and immune response indices, such as selected immune response indices related to blood levels of *Nr4a1 and Nr4a2* mRNA

Dependent variables	Independent variables	Unstandardized coefficient	df	R^2^	F	*P* values
IL-17	*Nr4a1* mRNA	-44.64	1, 8	0.68	14.7	0.006^**^
	*Nr4a2* mRNA	14.77	1, 8	0.22	2.0	0.204
IFN-γ	*Nr4a1* mRNA	-10.70	1, 8	0.51	7.4	0.030^*^
	*Nr4a2* mRNA	-0.96	1, 8	0.01	0.1	0.778
IL-10	*Nr4a1* mRNA	2.19	1, 8	0.45	5.7	0.048^*^
	*Nr4a2* mRNA	-0.53	1, 8	0.08	0.6	0.467
*Foxp3* mRNA	*Nr4a1* mRNA	-0.02	1, 8	0.00	0.0	0.901
	*Nr4a2* mRNA	0.13	1, 8	0.47	6.1	0.043^*^
*Lag3* mRNA	*Nr4a1* mRNA	0.14	1, 8	0.16	1.3	0.289
	*Nr4a2* mRNA	0.05	1, 8	0.05	0.4	0.554
*RORγt* mRNA	*Nr4a1* mRNA	-0.03	1, 8	0.01	0.1	0.824
	*Nr4a2* mRNA	0.06	1, 8	0.08	0.6	0.474
*IL-23R* mRNA	*Nr4a1* mRNA	-0.62	1, 8	0.40	4.7	0.068
	*Nr4a2* mRNA	0.38	1, 8	0.05	0.4	0.043^*^
*Nr4a1* mRNA	Prednisolone	-0.13	1, 8	0.65	12.9	0.009^**^
	Pembrolizumab	-0.15	1, 8	0.77	19.8	0.004^**^
*Nr4a2* mRNA	Prednisolone	0.08	1, 8	0.08	0.62	0.457
	Pembrolizumab	0.00	1, 8	0.00	0.00	0.983
*NLR*	Nr4a1 mRNA	-0.46	1, 8	0.18	1.578	0.249
	Nr4a2 mRNA	0.07	1, 8	0.01	0.089	0.774
*PLR*	Nr4a1 mRNA	-3.09	1, 8	0.00	0.021	0.889
	Nr4a2 mRNA	-6.13	1, 8	0.04	0.251	0.632

The neutrophil-to-lymphocyte ratio (NLR) and platelet-to-lymphocyte ratio (PLR) were used as predictive factors for serious immune-related adverse events. The plasma concentrations of pembrolizumab and cytokines, such as IL-17, IFN-γ, and IL-10, were evaluated by ELISA. Target mRNAs, such as *Nr4a1, Nr4a2*, *IL-23 receptor (IL-23R), Foxp3, RORγt, and Lag3,* were evaluated by RT‒qPCR methods with extracted total RNA from peripheral blood mononuclear cells. *p < 0.05, **P < 0.001

**Fig. 2:**
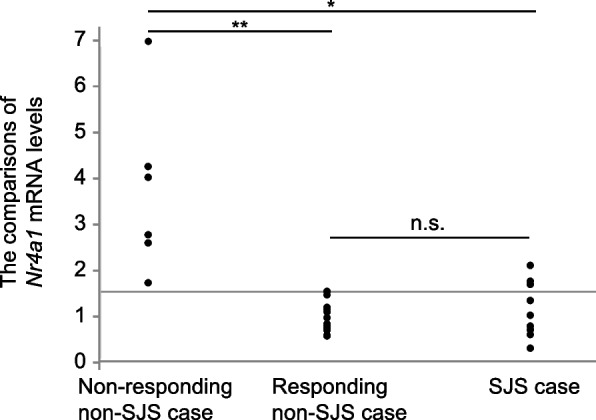
Comparisons of *Nr4a1* mRNA expression levels. Individual levels were measured relative to the basal level in SJS cases. Levels in an SJS case (*n* = 9) were compared between two types of single non-SJS prednisolone-free controls, a nonresponding non-SJS progressive disease case (*n* = 6) and a responding non-SJS case for pembrolizumab (*n* = 17), using the Steel–Dwass test. Blood samples were drawn during hospitalization and after discharge and used for the evaluations. *p < 0.05, **P < 0.001

*Nr4a1* mRNA levels during the observation period in the current SJS case were compared with those in two types of single non-SJS controls without prednisolone administration with the same carcinoma background. Nonparametric comparisons among the three groups were performed using the Steel–Dwass test. One was a nonresponding case of pembrolizumab progressive disease: 70 years old, male, cT1bN2M1b, stage IV; treatment was discontinued after the third course due to tumor growth on Day 63, with 6 points of blood collections. Another was a responding case: 69 years old, male, cT2aN3M0, stage IIIb; continued treatment every 21 days over Day 378, with 17 points of blood collections until Day 378. Plasma concentrations of pembrolizumab on Day 63 for the responding case and nonresponding case were 8.4 and 8.7 µg/mL, respectively. As shown in Fig. 2, *Nr4a1* mRNA levels in the SJS case and the responding non-SJS case were significantly lower than those in the nonresponding non-SJS case (*p* = 0.011 and *p* = 0.001). No significant difference in levels was observed between the SJS case and responding non-SJS case.

## Discussion and conclusion

Known predictive factors of serious irAEs, such as NLR and PLR [5, 6], were well indicative of the current occurrence of serious irAEs, i.e., SJS in this case. The basal value was observed to be higher than the NLR cutoff value of 3 and lower than the PLR cutoff value of 180 [5, 6]. However, the eosinophil and lymphocyte counts were lower than the respective cutoff values of 240/µL and 2000/µL, which were within acceptable limits [15]. IL-10 and *RORγt* mRNA were independently and significantly strongly involved in the increase in NLR and the decrease in PLR, respectively (Table 1). These results suggest that increased NLR is associated with decreased function of Tregs responsible for IL-10 production, which is required for suppressing excessive immune responses, and decreased PLR is associated with enhanced transcription of RORγt, the master transcription factor of Th17.

**Fig. 3:**
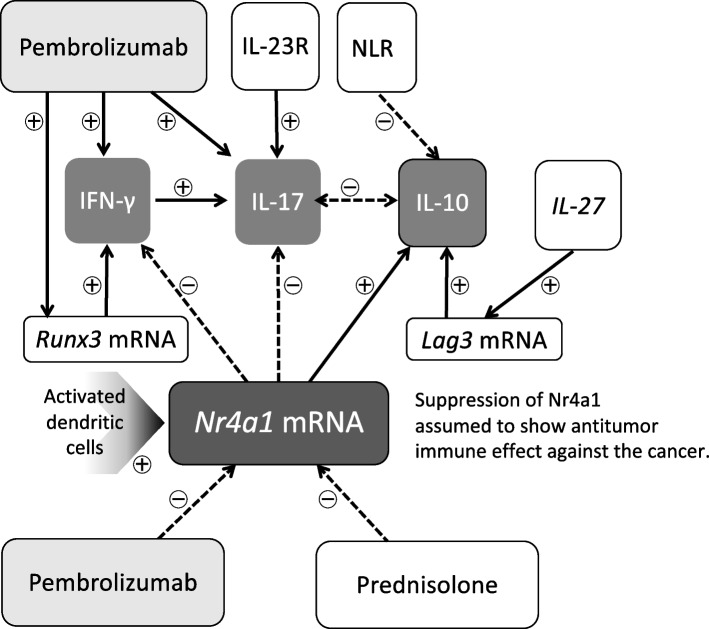
Diagram of the assumed relationship of indices in the SJS case. Possible facilitating relationships are indicated by arrows, and inhibitory relationships are indicated by dotted arrows based on a single regression analysis. Acute inductions of IFN-γ, IL-17 and suppression of IL-10, supposed to be influenced by the suppressed *Nr4a1* mRNA, assumed to contribute to the onset of SJS. Pembrolizumab and prednisolone assumed to contribute to the suppression of Nr4a1 in this case

In Th17 cells, which are involved in SJS, increased IL-17 in blisters and blood derived from Th17 cells has been reported [16]. In the current case, the increments of IL-17 and the deficiency of IL-10 following the transient increase in IFN-γ were observed at the onset of SJS (Fig. 1). Due to the limited number of measurement points, only a single regression analysis was possible, but an assumed relationship diagram based on significant results (Tables 1 and 2) is shown in Fig. 3. There was no relationship observed between IFN-γ, IL-17 and IL-23, an upstream regulator of memory Th1 and Th17 cells [14], which indicates no direct contributions of IL-23 to the onset. The characteristic increase in IL-17 was strongly affected by an increase in IFN-γ, and the increase in IFN-γ was strongly influenced by a decrease in the expression of *Nr4a1* mRNA and an increase in the expression of *Runx3* mRNA with a significantly high contribution rate. Loss of Nr4a1 induced Runx3 expression, resulting in the induction of cytotoxic CD8 ^+^ T cells in peripheral blood [11]. Thus, IFN-γ-producing CD8 ^+^ T cells were suggested to be significantly and rapidly induced, especially in the acute phase of SJS, even though cells could not be analyzed directly.

In this case, the decrease in *Nr4a1* mRNA levels was shown to be strongly involved in the induction of IL-17 as well as IFN-γ. Furthermore, this decrease in *Nr4a1* mRNA was very strongly influenced by the administration of prednisolone in addition to the unbound plasma concentration of pembrolizumab. Thus, consistent with the amelioration of excessive increases in IFN-γ and IL-17 during the SJS improvement phase by steroid therapy and the disappearance of ICI, there was also an amelioration of Nr4a1 levels.

The current case shows that the increase in *IL-23R* mRNA, not *RORγt* mRNA, was strongly involved in the induction of IL-17. The induction of *IL-23R* mRNA was strongly affected by increased *Nr4a2* mRNA levels but not by the concentration of ICI. Nr4a2 is required for Th17 differentiation and maintenance through the production of IL-21 without increasing the expression of RORγt. The master transcription factor for Th17 differentiation reportedly upregulates the expression of IL-23R, which is required for the increase and stabilization of Th17 [10, 14]. Therefore, induced Nr4a2 might have promoted the production of IL-17 through the upregulation of IL-23R on Th17 cells in this case. Functional Tregs are required for suppressive control of the excessive function of Th17 cells.

In this case, the allelic variant of HLA had not been evaluated. The allelic variant of HLA only identifies susceptibility to drug eruption, not the number of Tregs in PBMCs, but dysfunction of Tregs determines the type of disease, including SJS [17]. In general, Treg dysfunction due to infection has been reported prior to the onset of SJS [18]. Treg dysfunction seems to be consistent with our case. Chronic inflammation from lung cancer or prior chemotherapy can cause dysfunction of Foxp3^+^ Tregs, despite the absence of infection in the current case. Nr4as are essential for Treg development, suppressive functions, maintenance of Foxp3 expression, and suppression of cytokines [10, 19]. Nr4a2 is thought to be the main identity of Foxp3, and Nr4a1-deficient Tregs easily lose Foxp3 due to their plasticity [19, 20]. In this case, *Nr4a2* mRNA was strongly involved in the induction of *Foxp3* mRNA; however, this *Foxp3* mRNA was not involved in the induction of IL-10 levels, and conversely, the reduction of *Nr4a1* mRNA was strongly involved in the IL-10 deficiency. Thus, the effect of Nr4a1 reduction on Foxp3 plasticity may be greater than the effect of Nr4a2-mediated Foxp3 maintenance, leading to reduced IL-10 production due to dysfunction of Foxp3 + Tregs during SJS. In addition, a decrease in Nr4a1 may, albeit indirectly, affect the increase in NLRs via its effect on IL-10.

On the other hand, IL-27 was promotively involved in the induction of *Lag3* mRNA in this case. IL-27, with anergy to T-cell receptors, induces Foxp3^−^Lag3^+^ Tregs and peripheral inducible Tregs, which contribute significantly to IL-10 production [21]. Based on the study, the increase in IL-10 after steroid treatment was strongly influenced by induced expression of *Lag3* mRNA and not by the Nr4a family. Therefore, peripherally induced Tregs via Lag3 induction by IL-27, which was produced by activated dendritic cells (DCs), are thought to be responsible for this delayed induction of IL-10 around on Day 175. There was no direct involvement between IL-10 and IL-27 observed. We speculate that this was due to the time lag required for cytokine-mediated effector T cell interactions, unlike prompt responsive mRNA expression.

An inhibitory regulatory mechanism of Th17 other than Treg under the coexistence of TGF-β and IL-6 has been reported; Foxp3 binds to RORγt, the master transcription factor of Th17, and suppresses the expression and activation of RORγt under this coexistence [22]. In the present case, such coexistence was observed, but no significant relationship was found between TGF-β, IL-6, *Foxp3* mRNA and *RORγt* mRNA.

The slow but highly individualized disappearance of pembrolizumab [12] and the presence of this neutralizing antibody against pembrolizumab [23], which was not measured in this analysis, may have contributed to the long-term remission after discontinuation of this treatment. The level of *Nr4a1* mRNA was reduced below the basal level observed until Day 77, when the plasma concentration of unbound pembrolizumab was measurable. The plasma concentration of unbound pembrolizumab indicated very strong promotive effects on the induction of IFN-γ and *Runx3* mRNA and suppressive effects on the expression of *Nr4a1* mRNA, based on the present results. The reduction in Nr4a1 may have been initially affected by pembrolizumab and may have continued to promote an increase in IFN-γ and Runx3 expression. Furthermore, steroid treatment may have been involved in maintaining the Nr4a1 levels below the basal levels once reduced by pembrolizumab. The increase in *Nr4a1* mRNA around Day 175 may be due to activation of TCR following antigen presentation by DCs [10]. We also speculate that the gradual decrease in pembrolizumab and prednisolone, which was shown to have an inhibitory effect on Nr4a1, partly contributed to the release of suppression of Nr4a1 at that time. Regarding the cause of DC activation, the possibility of infection was ruled out, as there was no fever or increase in neutrophils or CRP (data not shown). The details are unknown, but some antigens attributed to tissue damage and repair by SJS are thought to enhance expression of MHC class II on DCs, resulting in activation.

The *Nr4a1* mRNA levels during the observation period in the current SJS cases were variable but, as in the responding case, were significantly lower than those in the nonresponding case (Fig. 2). Suppression of Nr4a1 leads to upregulation of functional cytotoxic CD8^+^ T cells and reduction of functional CD4^+^ Tregs, which may promote antitumor immunity in the tumor microenvironment [11]. These favorable immune responses were induced and may have led to complete remission of lung cancer in the current SJS case.

A single dose of pembrolizumab induced SJS, which was successfully treated with steroids, resulting in a complete remission of lung cancer. The immune response involving Nr4a1 suppression assumed to show a strong favorable antitumor immune effect against the cancer, despite causing severe irAEs. This may have been due to the synergism of the single but long-lasting effect of pembrolizumab and the appropriate intervention with steroids.

The current case demonstrates the preliminarily clinical utility and significance of evaluating Nr4a-related indices in the peripheral blood as the possible clinical covariates and constitutes a milestone for future research in terms of both efficacy and side effects.

### Limitation

A single rare case was reported. Due to the limited number of blood collections during the observation period, only a single regression analysis was performed. Multiple regression analysis followed by a single regression analysis with more points is needed to clarify the details. Further validation with more cases is needed to confirm the possible usefulness and significance of the current findings.

## Supplementary Information

**Additional file 1: Supplementary Table 1.** List of primers for RT‒qPCR.

## Data Availability

All data generated or analyzed during this study are included in this published article and are also available from the corresponding author upon reasonable request.

## Abbreviations

ICI: Immune checkpoint inhibitor
SJS: Stevens-Johnson syndrome
irAE: Immune-related adverse event
NLR: Neutrophil to lymphocyte ratio
PLR: Platelet to lymphocyte ratio
Treg: Regulatory T cell
PBMCs: Peripheral blood mononuclear cells
Nr4a: Nuclear receptor subfamily 4 group A
Foxp: Forkhead box protein P
RORγt: Retinoic acid receptor-related orphan receptor-γt
Runx: RUNX Family Transcription Factor
Lag 3: Lymphocyte activation gene 3
IFN-γ: Interferon-γ
TGF-β: Transforming growth factor-β
IL: Interleukin
IL-23R: Interleukin 23 Receptor
