# Supplementary material for: A case report involving suppressed nuclear receptor transcription factors 4a1 and Stevens-Johnson syndrome induced by a single dose of pembrolizumab and successfully treated with early steroid administration, resulting in complete remission of stage III lung cancer
Source: J Pharm Health Care Sci. 2022 Dec 5;8:29. doi: 10.1186/s40780-022-00261-y (PMC9720965; doi:10.1186/s40780-022-00261-y)
Supplement: Supplementary file 1 — Additional file 1: Supplementary Table 1. List of primers for RT‒qPCR. [file 40780_2022_261_MOESM1_ESM.docx]

Supplementary Table 1.

List of primers for RT‒qPCR

Gene name Forward primer Reverse primer

*Nr4a1* 5'-CACAGCTTGCTTGTCGATGTC -3' 5'- ATGCCGGTCGGTGATGAG -3'

*Nr4a2* 5'-TATTCCAGGTTCCAGGCGAA-3' 5'- GCTAATCGAAGGACAAACAG-3'

*Foxp3* 5’-GAGAAGCTGAGTGCCATGC-3’ 5’-GTATCCAGTGCGTGTCGTGG-3’

*RORγt*　 5'-GCAGCGCTCCAACATCTTCT-3' 5'-ACGTACTGAATGGCCTCGGT-3'

*Lag3* 5'-TCACAGTGACTCCCAAATCCT-3' 5'-GCTCCACACAAAGCGTTCTT-3'

*Runx3* 5'-GGCAATGACGAGAACTACTCCG-3’ 5’-GATGGTCAGGGTGAAACTCTTCC-3’

*IL-23R* 5'-AACAGCAATGTTGTGAAAATGC-3' 5'-AGGCTTGTGTTCTGGGATGA-3'

*HPRT*  5'-TATGGACAGGACTGAACGTC-3' 5'-CATCAATGTGATGGCCTCCC-3’
